# Velocity Sensor for Real-Time Backstepping Control of a Multirotor Considering Actuator Dynamics

**DOI:** 10.3390/s20154229

**Published:** 2020-07-29

**Authors:** Walter Alejandro Mayorga-Macías, Luis Enrique González-Jiménez, Marco Antonio Meza-Aguilar, Luis Fernando Luque-Vega

**Affiliations:** 1 Department of Electronics Systems and Computing, Instituto Tecnológico y de Estudios Superiores de Occidente, ITESO AC, Tlaquepaque 45604, Jalisco, Mexico; ng700810@iteso.mx (W.A.M.-M.); mar_ant8@hotmail.com (M.A.M.-A.); 2 Centro de Investigación, Innovación y Desarrollo Tecnológico CIIDETEC-UVM, Universidad del Valle de México, Tlaquepaque 45601, Jalisco, Mexico; luis.luque@uvmnet.edu

**Keywords:** actuator dynamics, angular velocity sensors, backstepping, nonlinear controller, sliding mode differentiator, unmanned aerial vehicle

## Abstract

A real-time implementation of a control scheme for a multirotor, based on angular velocity sensors for the actuators, is presented. The control scheme is composed of two loops: an inner loop for the actuators and an outer loop for the unmanned aerial vehicle (UAV). The UAV control algorithm is designed by means of the backstepping technique and a robust sliding mode differentiator, and the actuator control strategy is based on a standard proportional-integral-derivative (PID) controller. A robust exact differentiator, based on high order sliding modes, is used to estimate the complex derivatives present in the proposed control law. As the measurements of the propeller’s angular velocities are required for the control law, velocity sensors are mounted in the axles of the rotors to retrieve them and a signal conditioning stage is implemented. In addition, dynamical models for the actuators of the aircraft were calculated by means of transfer functions obtained via experimental measurements in a test bench developed for this purpose. This test bench permits to characterize the parameters of the transfer functions by comparing the forces computed using the nominal parameter to the measured forces. To this end, it is assumed that the loads in the actuators of the vehicle are insignificant during flight. The effectiveness of the proposed sensor, its signal conditioning, and the overall control scheme are validated by means of simulation results and real-time experiments.

## 1. Introduction

An unmanned aerial vehicle (UAV) is an aircraft with no aircrew, which is replaced by a control computer system and radio-link for remote controllability or autonomous flight. The applications of UAVs cover diverse technology areas from monitoring and surveillance to transportation, which influence research fields as computer vision, mechanical design and automatic control. Moreover, from a control point of view, a UAV is a complex, nonlinear, highly coupled dynamical system with multiple inputs and outputs that is usually disturbed by diverse environmental factors as wind, air density variations, and obstacles. Hence, in order to implement a UAV flight controller in real-time, it is important to first validate the designed control scheme using simulation results and, then, implement it in an electronic embedded system. In particular, multirotors are a type of UAV characterized by easy construction and control.

A great diversity of research works oriented to the control for multirotors can be found in the literature, as shown in [1,2]. The control scheme approaches include classic control schemes as PID (Proportional-Integral-Derivative) [3,4]; LQR (Linear Quadratic Regulator) [5]; and MPC (Model Predictive Control) [6,7], the main feature of which is the ability to predict behavior of the controlled system to react accordingly; nevertheless, its robustness depends on the accuracy of the obtained model of the system, which usually is based on empirical experimentation. Moreover, standard control techniques as backstepping methods have been merged with more advanced control algorithms to add desired control features in the closed loop system as in [8,9,10,11]. Amongst these advanced nonlinear control strategies, the so-called intelligent control schemes based on neural networks [12,13] and fuzzy logic [14,15] have been implemented to provide an extension of linear control techniques for more operating points of the plant, or to include the capacity of generalize the model of a subsystem of the UAV for control purposes. These are attractive approaches for controlling multirotors, however, their implementation is complex and computationally expensive which are undesirable features when a real-time implementation of the control scheme is required. Additionally, another desired characteristic for a multirotor controller is robustness as the vehicle is usually disturbed by external factors during a normal real-time operation. In that regard, the adaptive control approach has been used to robustly control multirotors in different flight modes as in [16,17]. But, when it comes to robustness, the sliding mode theory [18] has demonstrated excellent results for quadrotor controllers as in [9,11,19,20]. Nonetheless, it is usually accompanied by an undesirable phenomenon called chattering, which is characterized by high frequency components in the control signal, affecting negatively the actuators of the controlled system.

On the other hand, the standard instrumentation of multirotors includes a global positioning system (GPS) sensor module to retrieve the position of the vehicle, and an inertial measurement unit (IMU) to obtain the angular and linear velocities of the UAV. However, the angular velocities of the axles of the actuators are usually discarded even when this information could permit to obtain the generated thrust, consumed current, and fault detection in the actuators [21]. This could enable the development of applications for accurate range and endurance estimation, model-based control design, real-time aerodynamic drag estimation and propeller performance tracking. Some works have been oriented to implement sensors to monitor the thrust generation during flight in multirotors using force sensors [22,23,24]; and model-based estimations [25]. Also, the rotors angular velocities can be fused with other sensors signals as in [26] where rotor speeds where merged to the accelerometer’s outputs using a Kalman filter for attitude stabilization of the UAV. In addition, several algorithms have been used for speed control of Brushless Direct Current (BLDC) motors but, amongst them, the PID control features ease implementation and avoids the necessity of modelling the actuator, which tends to be troublesome due to the parameter identification. For instance, in [27,28] a PID algorithm combined with fuzzy control was used for reference tracking of the angular velocity of a BLDC motor. In [29], an adaptive PID control algorithm was designed and in [30] an adaptive fuzzy PID control scheme was used, both for the same purpose. Finally, the PID control has also been combined with more complex intelligent algorithms as deep neural networks. In [31] such a controller was designed for the speed control of a BLDC motor and its stability analysis was presented. In summary, the best performance for a multirotor control scheme is usually obtained by combining control techniques that add up features as disturbance robustness, energy efficiency, transient characteristics, and steady state error. However, as the objective of this work is to present the performance of a control scheme with the inclusion of velocity sensors for the actuators, we aimed to the simplicity and ease embedded implementation of the proposed control strategy. Due to that, in this work, a backstepping controller is proposed for the trajectory tracking of the attitude and altitude of a quadrotor. The overall control scheme considers an inner loop PID controller for the actuators (BLDC motors) of the multirotor and the implementation of velocity sensors for their axles. First, the dynamic model of the UAV and its actuators is obtained. It is worth to note that insignificant loads in the actuators of the vehicle are assumed, i.e., small accelerations of the multirotor and negligible external disturbances during flight. Then, the backstepping control algorithm is designed, and simulations results for the closed loop system are shown. Afterwards, real-time experiments are developed, and their results are documented and discussed. Finally, the conclusions of the presented work are outlined.

## 2. UAV’S Dynamical Model

**Figure 1:**
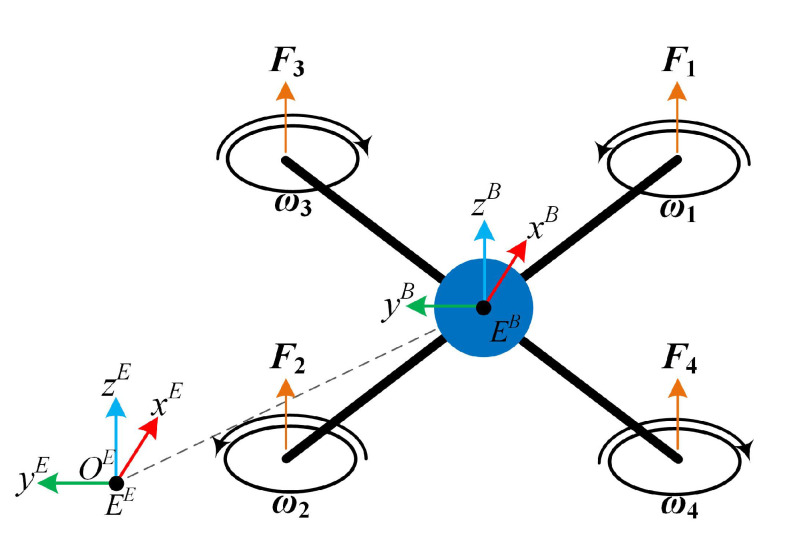
The unmanned aerial vehicle’s (UAV’s) configuration and related variables.

A graphical representation of the multirotor considered in this work is depicted in Figure 1. The position of the vehicle is defined by its Cartesian coordinates (x,y,z) which corresponds to the translation vector between the origins of and inertial reference system $x^{E},y^{E},z^{E}$ and a coordinated system $x^{B},y^{B},z^{B}$ fixed to the centre of gravity of the UAV. Therefore, the orientation of the vehicle is defined by the Euler angles between these two coordinate systems: roll (ϕ), pitch (θ) and yaw (ψ). Defining a state vector as $X={\left[\phi ,\dot{\phi },\theta ,\dot{\theta },\psi ,\dot{\psi },z,\dot{z},x,\dot{x},y,\dot{y}\right]}^{T}$, the state space representation of the vehicle can be obtained as:

(1) $$\dot{X}=\left[\begin{array}{c} x_{2}\\ f_{2}+b_{2}U_{1}\\ x_{4}\\ f_{4}+b_{4}U_{2}\\ x_{6}\\ f_{6}+b_{6}U_{3}\\ x_{8}\\ f_{8}+b_{8}U_{4}\\ x_{10}\\ b_{10}U_{1}\\ x_{12}\\ b_{12}U_{1} \end{array}\right]$$

where $f_{2}=-g$, $f_{4}=x_{4}x_{6}\frac{I_{y}-I_{z}}{I_{x}}-\frac{J_{r}}{I_{x}}x_{4}\Omega$, $f_{6}=x_{2}x_{6}\frac{I_{z}-I_{x}}{I_{y}}+\frac{J_{r}}{I_{y}}x_{2}\Omega$, $f_{8}=x_{2}x_{4}\frac{I_{x}-I_{y}}{I_{z}}$, $b_{2}=\frac{c_{1}c_{3}}{m}$, $b_{4}=\frac{l}{I_{x}}$, $b_{6}=\frac{l}{I_{y}}$, $b_{8}=\frac{l}{I_{z}}$, $b_{10}=\frac{c_{1}s_{3}c_{5}+s_{1}s_{5}}{m}$, $b_{12}=\frac{c_{1}s_{3}s_{5}-s_{1}c_{5}}{m}$; with $I_{x}$, $I_{y}$, and $I_{z}$ as the moments of inertia of the multi-rotor frame body, $J_{r}$ the total inertia, *g* as the gravity acceleration, *m* as the total mass of the aircraft, $\Omega =\omega_{2}+\omega_{4}-\omega_{1}-\omega_{3}$, $\omega_{i}$ as the angular velocity of the *i*th rotor of the vehicle, $c_{i}=\cos x_{i}$, $s_{i}=\sin x_{i}$, and $X={\left[x_{1},\ldots ,x_{12}\right]}^{T}$. The variables $U_{1}$, $U_{2}$, $U_{3}$, and $U_{4}$ are given by

(2) $$\left[\begin{array}{c} U_{1}\\ U_{2}\\ U_{3}\\ U_{4} \end{array}\right]=M\left[\begin{array}{c} \omega_{1}^{2}\\ \omega_{2}^{2}\\ \omega_{3}^{2}\\ \omega_{4}^{2} \end{array}\right]$$

where

$$M=\left[\begin{array}{cccc} b & b & b & b\\ p & -p & -p & p\\ q & -q & q & -q\\ d & d & -d & -d \end{array}\right]$$

with $b=C_{T}\rho D^{4}$ as the thrust factor, *p* as the thrust factor seen from the *x* axis, *q* as the thrust factor seen from the *y* axis, and $d=(C_{D}\rho A)/2$ as the drag factor of the propellers, where $C_{T}$ is the thrust coefficient, ρ is the air density, *D* is the propeller diameter, $C_{D}$ is the drag coefficient, and *A* is the cross-section of movement.

### 2.1. Actuators Dynamical Model

**Figure 2:**
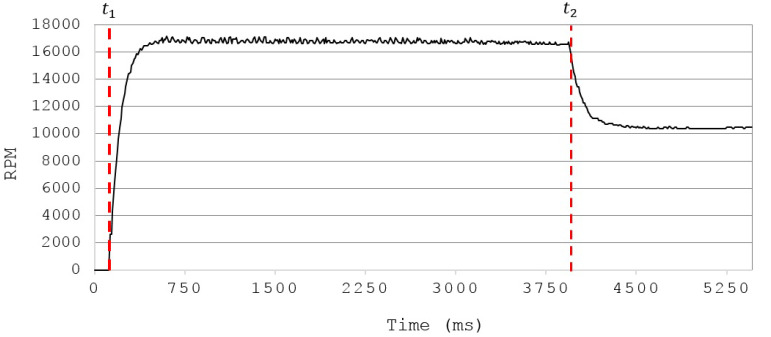
Step response of the actuators for transfer function characterization.

The actuators of the vehicle correspond to brushless DC (BLDC) motors that are controlled in open loop by electronic speed controllers (ESC) by means of suitable pulse-width modulation (PWM) signals generated by the electronic control unit. The dynamical model of this type of motor consist of a set of 5 ordinary differential equations and diverse parameters as electrical resistance and inductance, inertia, friction and back EMF (electro-mechanical force). In order to avoid the cumbersome characterization of these parameters, we propose to obtain their approximated transfer functions in an experimental manner, defining the duty cycle of the PWM signal and the rotor’s angular velocity as its input and output, respectively. The methodology consists of introducing a test input signal to the real motor and measuring the generated angular velocity. The test input signal was defined as a step signal of 100% of duty cycle at $t_{1}=120$ ms, and 50% of duty cycle at $t_{2}=3950$ ms. The experimental measurements are shown in Figure 2.

Then, this data is used to obtain an estimated transfer function by means of the System Identification Toolbox from Matlab [32]. Several pole-zero configurations were tried and the best result, with 95.6% accuracy, corresponded to the estimated transfer function $G_{m}\left(s\right)$ defined as:

(3) $$G_{m}\left(s\right)=\frac{716.6s^{2}+1.377\times 10^{5}s+7066}{s^{2}+11.16s+0.4724}$$

### 2.2. Dynamical Parameter Characterization

**Figure 3:**
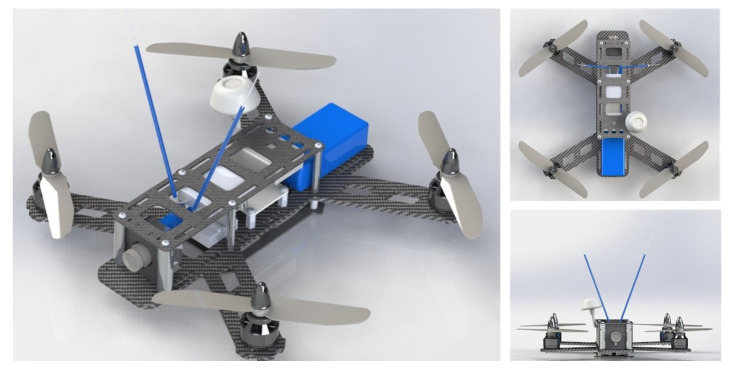
Top (**top-left**), isometric (**top-right**) and front (**bottom**) views of the 3D model of the quadrotor obtained in a Computer-Aided Design (CAD) software.

To determine completely the dynamic model of the quadrotor defined by Equations (1)–(3), the mechanical and electric parameters of the UAV must be characterized. The mechanical part of these parameters was obtained by means of a 3D model of the system designed in a computer-aided design (CAD) software [33], which is shown in Figure 3.

**Figure 4:**
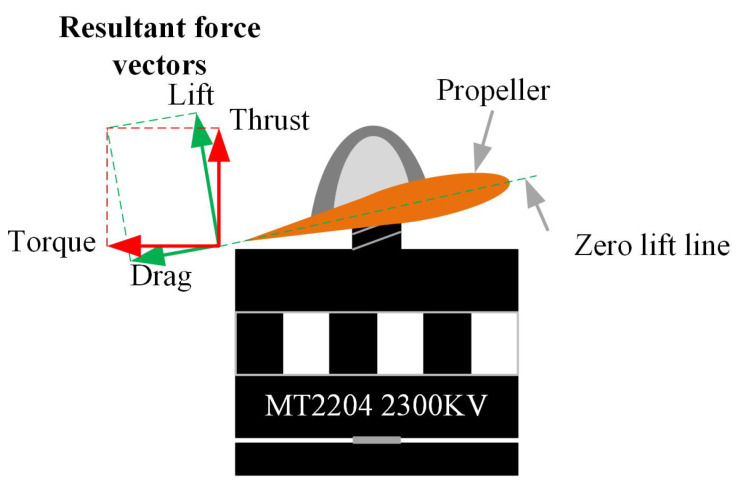
Force vectors produced by the propellers.

From this model, nominal values for the inertia terms $I_{x},I_{y},I_{z}$, the mass *m*, and length *l* were defined. On the other hand, the terms *b* and *d* are related to the drag and thrust forces generated by the propellers, these forces are depicted in Figure 4.

**Figure 5:**
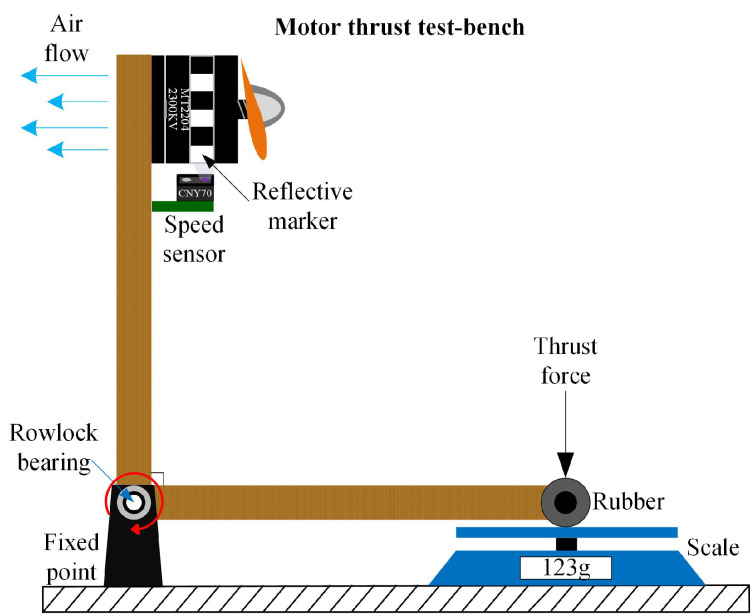
Thrust force test-bench for obtaining the thrust coefficient.

**Figure 6:**
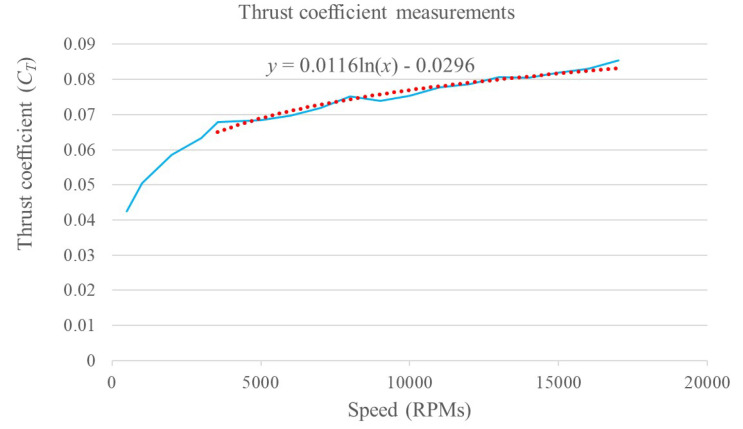
Thrust coefficient graph based on EMAX MT2204 2300KV motor thrust force measurements.

**Figure 7:**
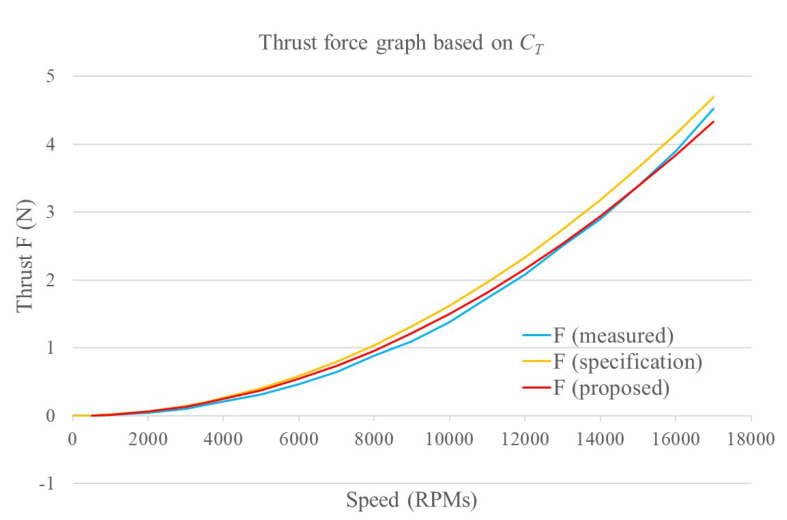
Thrust force graph from the thrust coefficient at different motor speeds using the measured coefficient, the motor manufacturer coefficient, and the proposed coefficient.

In addition, these parameters are defined by the following formulas:

(4) $$b=C_{T}\rho n^{2}D^{4}$$

(5) $$d=\frac{C_{D}\rho Av^{2}}{2}$$

where *D* is the propeller diameter in meters, *n* is the propeller/motor speed in rev/s, ρ is the air density in kg/m${}^{3}$, $C_{T}$ the thrust coefficient, *A* is the cross-section of the movement in m${}^{2}$, *v* is the drone velocity in m/s, and $C_{D}$ is the drag coefficient. The terms ρ, *D*, and *A* are assumed constant and approximated by nominal values. The terms *n* and *v* are measured by the UAV sensors and, in particular, the propeller velocity *n* sensor will be explained in detail in a following section. The remainder terms $C_{D}$ and $C_{T}$ are indicated as nominal values by some research works as in [9] where a thrust coefficient of $2.98\times 10^{-3}$ and a drag coefficient of $1.14\times 10^{-7}$ are presented, but there is no explanation or methodology to obtain these values, which is very important when it comes to motor control. Due to the fact that the cross-section area *A* facing the direction of movement of the drone is very difficult to calculate during flight, a fixed drag coefficient is proposed as $43\times 10^{-3}$. This value was adjusted experimentally during flight tests to obtain the best closed-loop performance. The thrust coefficient was obtained experimentally by means of a bench and a scale to create characteristic curves for the parameter. The overall test bench is shown in Figure 5. Its working principle is based on manually varying the propeller velocities and measuring the generated thrust by means of the scale. Figure 6 shows the obtained characteristic curve for $n-C_{T}$. From this, a $C_{T}$ value is proposed based on a minimal root mean squared error (RMSE) of the obtained curve and its correspondent function. In order to validate the obtained parameter, a comparison between the behavior generated by the manufacturer value, the proposed value and the measurements is presented in Figure 7. With a proposed $C_{T}=0.0817445$, a minimal RMSE of 0.077301668 *N* (0.7882576369 gf) is obtained. Compared to the measured values, this is relatively small with respect to the thrust force difference. Finally, this experimentally adjusted value for $C_{T}$ was used in this work, and the nominal value used in [9] for the drag coefficient $C_{D}$ was defined, as no experimental measurements related to this parameter were obtained.

## 3. Controller Design

**Figure 8:**
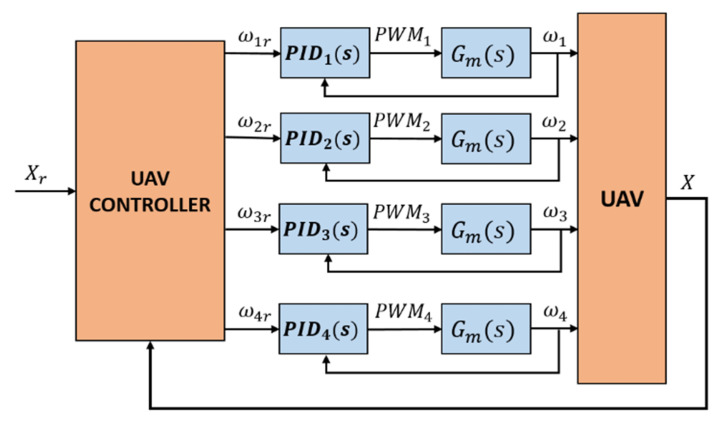
Overall proposed control scheme. Two control loops are depicted: the inner loop for the actuators and the outer loop for the UAV.

The control proposal is composed of two controllers: the UAV controller and the actuator controller. The input for the UAV controller is a reference vector $X_{r}$ that includes the altitude (*z*) and the Euler’s angles (ϕ, θ, ψ) of the multirotor, and its outputs are references values for the angular velocities of the actuators (or the propellers). The actuator controller is composed by four PID controllers, which receives the desired angular velocity and defines a correspondent PWM value as the input of the motor. This inner control loop is the same for all the four motors of the UAV. The overall control scheme is shown in Figure 8 and the UAV and actuator controller are defined in the following subsections.

### 3.1. UAV Controller

The control objective is the reference tracking for the orientation ($x_{1}=\phi$, $x_{3}=\theta$, $x_{5}=\psi$) and the altitude ($x_{7}=z$) of the UAV. Hence, it is assumed that the references variables $x_{1r}$, $x_{3r}$, $x_{5r}$ and $x_{7r}$ are known, as well as their derivatives. Then, in order to obtain a concise controller design procedure, four different sub-systems from Equation (1) are defined as:

(6) $$\begin{array}{cc} {\dot{x}}_{i} & =x_{i+1}\\ {\dot{x}}_{i+1} & =f_{i+1}+b_{i+1}U_{(i+1)/2},\;\;\;\;\;\mathrm{for}\;i=1,3,5,7 \end{array}$$

The error variables for these subsystems are given by $z_{i}=x_{ir}-x_{i}$, and their dynamics yields:

(7) $${\dot{z}}_{i}={\dot{x}}_{ir}-{\dot{x}}_{i}={\dot{x}}_{ir}-x_{i+1}$$

After that, a Lyapunov’s function is designed as $V_{i}=\frac{1}{2}z_{i}^{2}$ and its derivative results of the form:

(8) $${\dot{V}}_{i}=z_{i}{\dot{z}}_{i}=z_{i}\left({\dot{x}}_{ir}-x_{i+1}\right)$$

Then, the term $x_{i+1}$ is proposed as a virtual control term, which is designed as:

(9) $$x_{i+1}^{*}={\dot{x}}_{ir}+k_{i}z_{i},\;\;k_{i}<0$$

which will allow us to define the error variable for the next block. The term $x_{i+1}^{*}$ is the desired value for $x_{i+1}$, so, the error variable $z_{i+1}$ is defined as $z_{i+1}=x_{i+1}^{*}-x_{i+1}$ which can be used to re-shape the Equation (8) as ${\dot{V}}_{i}=z_{i}\left({\dot{x}}_{ir}-x_{i+1}^{*}+z_{i+1}\right)=-k_{i}z_{i}^{2}+z_{i}z_{i+1}$. Now, the dynamics for $z_{i+1}$ results of the form:

(10) $${\dot{z}}_{i+1}={\dot{x}}_{i+1}^{*}-{\dot{x}}_{i+1}={\ddot{x}}_{ir}+k_{i}\left({\dot{x}}_{ir}-x_{i+1}\right)-f_{i+1}-b_{i+1}U_{(i+1)/2}$$

where the control term $U_{(i+1)/2}$ will be designed for the convergence of Equation (10). To this end, the candidate Lyapunov’s function $V_{i+1}=V_{i}+\frac{1}{2}z_{i+1}^{2}$ is proposed, the derivative of which is given by:

(11) $$\begin{array}{cc} {\dot{V}}_{i+1} & =\;-k_{i}z_{i}^{2}+z_{i}z_{i+1}+z_{i+1}{\dot{z}}_{i+1}\\ & =\;-k_{i}z_{i}^{2}+z_{i}z_{i+1}+z_{i+1}\left[{\ddot{x}}_{ir}-f_{i+1}+k_{i}\left({\dot{x}}_{ir}-x_{i+1}\right)-b_{i+1}U_{(i+1)/2}\right] \end{array}$$

and the control term $U_{(i+1)/2}$ is designed as:

(12) $$U_{(i+1)/2}=b_{i+1}^{-1}\left[{\ddot{x}}_{ir}-f_{i+1}+k_{i}\left({\dot{x}}_{ir}-x_{i+1}\right)+z_{i}+k_{i+1}z_{i+1}\right]$$

with $k_{i+1}<0$. Using Equation (12), the term ${\dot{V}}_{i+1}$ yields:

(13) $${\dot{V}}_{i+1}=-k_{i}z_{i}^{2}-k_{i+1}z_{i+1}^{2}$$

which fulfils ${\dot{V}}_{i+1}\leq$0 and the convergence of $z_{i}$ and $z_{i+1}$ to zero is assured. Hence, the control objective is achieved.

### 3.2. Levant’s Differentiator

The terms ${\dot{x}}_{ir}$ and ${\ddot{x}}_{ir}$ of the control term Equation (12) are not defined. To avoid cumbersome computations for these expressions, a robust exact differentiator, proposed by Levant in [34,35], is used. The main problem with usual differentiator designs is the combination of exactness and robustness with respect to possible measurement errors and input noise. Moreover, if the input signal present disturbances, the differentiator’s output will be also disturbed. To overcome this, Levant’s differentiator provides the square of the maxima of the measured input signal from the base signal, reducing the rate error that can be present in the output [36], by means of a sliding mode control term. The Equations of the second order differentiator, used in this work, are given by

(14) $$\begin{array}{ccc} {\dot{z}}_{0} & = & -\lambda_{2}{\left|z_{0}-f\left(t\right)\right|}^{2/3}\mathrm{sign}\left(z_{0}-f\left(t\right)\right)+z_{1}\\ {\dot{z}}_{1} & = & -\lambda_{1}{\left|z_{1}-v_{0}\right|}^{1/2}\mathrm{sign}\left(z_{1}-v_{0}\right)+z_{2}\\ {\dot{z}}_{2} & = & -\lambda_{0}\mathrm{sign}\left(z_{2}-v_{1}\right) \end{array}$$

where f(t) is the input signal, $z_{i}$ is the estimation of the *i*th derivative of f(t), and $\lambda_{0}$, $\lambda_{1}$, $\lambda_{2}$ are differentiators parameters to be tuned. Hence, it can be used to obtain ${\dot{x}}_{ir}$ and ${\ddot{x}}_{ir}$ with $f\left(t\right)=x_{ir}$.

### 3.3. Actuator Controller

The UAV controller generates the desired values for $U_{1}$, $U_{2}$, $U_{3}$, and $U_{4}$. Afterwards, the desired values for the actuators’ velocities $\omega_{1r}$, $\omega_{2r}$, $\omega_{3r}$, and $\omega_{4r}$ can be obtained from Equation (2) as:

(15) $$\left[\begin{array}{c} \omega_{1r}^{2}\\ \omega_{2r}^{2}\\ \omega_{3r}^{2}\\ \omega_{4r}^{2} \end{array}\right]=M^{-1}\left[\begin{array}{c} U_{1}\\ U_{2}\\ U_{3}\\ U_{4} \end{array}\right].$$

From here, the error variables for the actuators’ controllers are defined as:

(16) $$e_{i}=\omega_{ir}-\omega_{i},\;\;i=1,2,3,4.$$

Finally, the equation of the PID controllers are defined as:

(17) $$PWM_{i}=k_{Pi}e_{i}+k_{Ii}\int e_{i}dt+k_{Di}\dot{e_{i}}$$

where the control gains $k_{Pi}$, $k_{Ii}$, and $k_{Di}$ are tuned heuristically. The $PWM_{i}$ signals correspond to the duty cycles of the ESC for each motor. It is worth to note that, as the motors can only rotate in one direction, the $PWM_{i}$ values are bounded by the interval [0,100]%.

## 4. Experimental Setup

The UAV used for the experiments is composed of a quadrotor QAV250 frame with MT2204-2300KV brushless motors and 6030 propellers. Each motor is driven by a 2-4S 12A electronic speed controller (ESC) with an 11.1 V 2200 mAh Lithium-Polymer battery as a power supply. The main control board of the vehice is a PIXHAWK PX4 2.4.6 which is an STM32F427 microcontroller based board that uses an STM32F100 as a failsafe system. It includes an L3GD20H 3-axis gyroscope sensor, an LSM303D 3-axis accelerometer, an MPU6000 inertial measurement unit (IMU), and an MS5611 barometer. The board provides 1 ADC channel, 1 CAN port, 1 I2C port, 5 UART ports, and supports up to 14 pulse protocol outputs. In order to implement the proposed controller for the real-time experiments, it is necessary to obtain measurements of the rotors angular velocities; nonetheless, there are no available ports in the main board. Hence, a daughter board that allows capturing of the rotors sensors signals was included. It is based on an ATMEGA328/P microcontroller, which is capable of running at a frequency of 16 MHz, has 8 ADC channels available with up to 10-bit precision, and a universal synchronous and asynchronous receiver-transmitter (USART). As a result, it can be used to sense up to 8 analog sensors and transmit the data through serial communication to the main board.

### 4.1. Angular Velocity Sensor for the UAV Rotors

**Figure 9:**
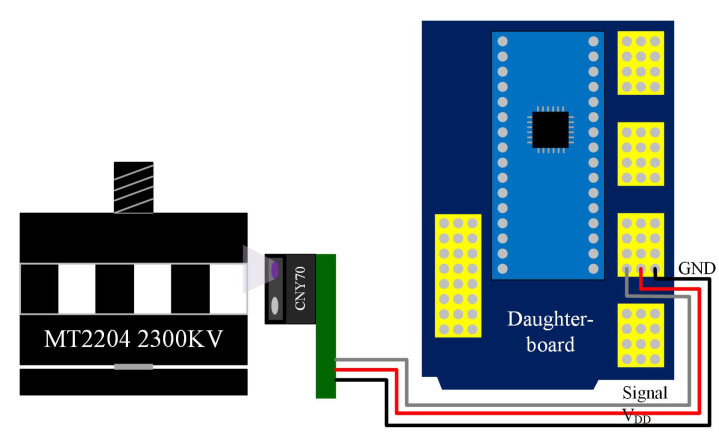
Propeller’s velocity sensor implementation based on a CNY70 reflective sensor.

As part of the inner loop controller for the motors, a sensor for the propeller’s velocities must be designed. Since the motors used in this work do not have any type of encoder, an intermittent black and white band is attached to the motor case acting as encoder. Additionally, CNY70 reflective sensors are used for capturing the light intensity change in the band when the motor is rotating, generating a pulse frequency that feeds a pin voltage level change interrupt in a daughter board. The interconnection between the reflective sensor, the motor and the daughter board are presented in Figure 9.

This daughter board is proposed to provide modularity to the firmware development and to implement the digital conditioning of the signals generated by the reflective sensors. The signal generated by the reflective sensors triggers an interruption in the daughter board. This interruption is time tracked with a timer and five different captures are obtained. The first capture takes a false pulse that was observed and is taken in the middle of a pulse generated (not at the rising or falling edge), and then the next four pulses are timer captured, having two complete periods observed, which are averaged to obtain better accuracy. The encoder black and white band attached has 40 alternating stripes within a motor revolution. To avoid overlap in the different interruption’s tasks, an interrupt scheduler routine is designed to assign execution status to the different sensing tasks. In that way, only one interruption is running at a time, avoiding the interrupt miss for the speed period capturing, which is extremely time sensitive.

### 4.2. Velocity Sensor’s Signal Conditioning

Due to the switching nature of the velocity sensor and the noisy characteristics of its optical transducer, the retrieved measurements presented undesired high frequency fluctuations. Hence, a digital filtering stage was designed in the daughter board. Two different proposals were implemented: a moving average (MA) filter and an exponentially weighted moving average (EWMA) filter. The first is a finite impulse response filter which consists in obtaining the average value of the last *m* samples of the measured signal according to the following Equation:

(18) $$y_{k}=\frac{\sum_{i=k-m}^{k}x_{i}}{k-m}$$

where $x_{k}$ is the current sample of the measured signal and $y_{k}$ is the current output of the filter. The latter filter is a recursive or infinite impulse response filter which is implemented by the following Equation:

(19) $$y_{k}=y_{k-1}+\alpha \left(x_{k}-y_{k-1}\right)$$

where α is the constant weight assigned to the filter. The performance comparison between both filters provided a solid ground to choose the more promising filter. The EWMA exposes a smaller RMSE (root-mean-square error) and provides smoother output behaviour as the input data becomes gradually less influential as it ages. On the other hand, MA treats each of the samples equally during the output computing but these are regarded as soon as the samples fall off the end of the number of elements considered in the filter. Therefore, the EWMA filter was selected to be used in this work.

### 4.3. Overall Controller Implementation

The global designed embedded control scheme was implemented in the main board of the vehicle using the model based approach as in [37,38]. It is characterized as a closed-loop controller where the control laws are computed based on the measured states of the vehicle as depicted in Figure 8. The stages of the implemented control strategy are described in the Algorithm 1. All the simulation and real-time experiments were carried out using the controller implementation described in this method.
**Algorithm 1:** Overall Controller Execution.
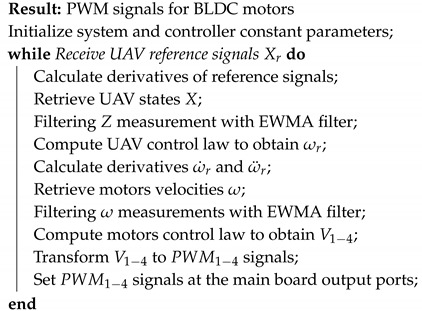



## 5. Results

The control proposal was implemented in simulation and real-time experiments considering the same control and system parameters aiming to analyse the results, which are presented in this section.

### 5.1. Simulation Results

The parameters used for the simulation experiments are listed in Table 1. These correspond to the parameters obtained by the methodology described in the previous sections. The control objective is to track the Euler angles (ϕ, θ, ψ) and the altitude (*z*) of the multirotor vehicle.

**Figure 10:**
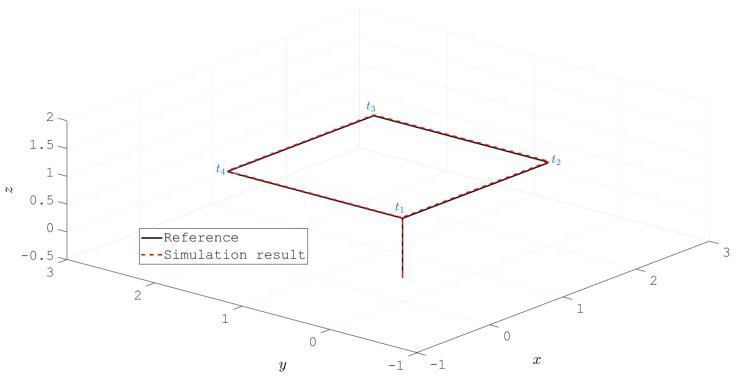
Position of the vehicle resulting from the attitude and altitude reference tracking.

**Figure 11:**
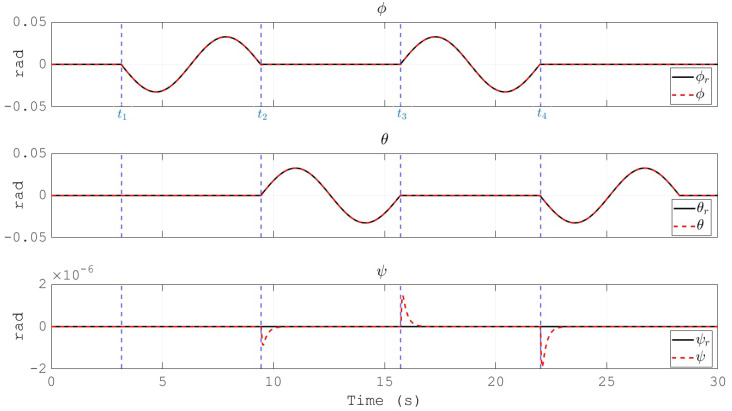
Euler angles of the multirotor and their references.

**Figure 12:**
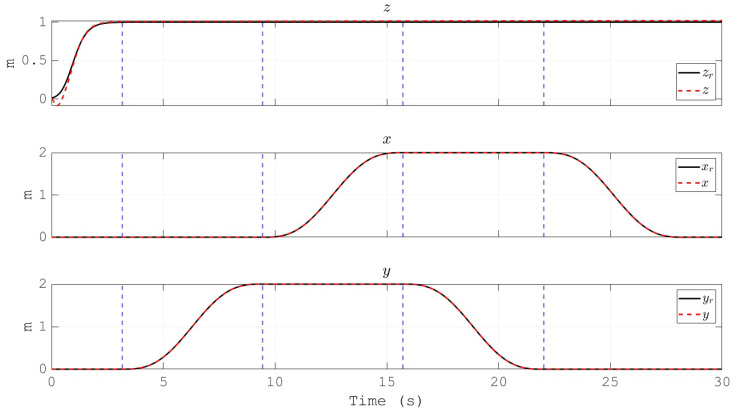
Cartesian coordinates of the position of the multirotor. Only the altitude *z* is tracking its reference $z_{r}$.

**Figure 13:**
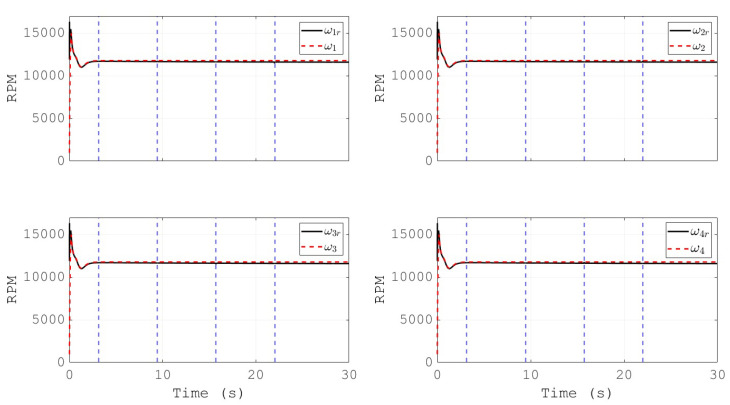
Commanded propellers velocities and their references.

The resulting trajectory of the UAV position describes a square path as depicted in Figure 10. It is worth to note that, as a stabilize flight mode is considered, the references received by the overall control scheme are for the attitude ($\phi_{r}$, $\theta_{r}$ and $\psi_{r}$) and altitude ($z_{r}$) of the vehicle. Hence, the square shaped position depicted in Figure 10 is a direct result of the definition for $\phi_{r}$, $\theta_{r}$ and $\psi_{r}$. The labels $t_{1}$, $t_{2}$, $t_{3}$ and $t_{4}$ indicate the instants where the trajectory reaches the corners of the square. For easy correlation, these timestamps are also indicated in Figure 11, Figure 12 and Figure 13 by means of vertical blue dashed lines. This is a direct result of the reference defined for the attitude of the UAV which is shown in Figure 11. Moreover, the references for the three angles are depicted as well. The tracking performance is satisfactory as the proposed control scheme obtains the convergence of the error variables to a small vicinity to zero.

Figure 12 depicts the generated Cartesian coordinates of the vehicle during the simulation, and for the case of the attitude signal the reference is also presented. The convergence of the altitude to its reference in a finite time can be noted. Furthermore, the angular velocities of the propellers, and their references, are shown in Figure 13. This behavior validates the effectiveness of the PID controllers for the actuators of the vehicle in the inner loop, as the tracking performance is satisfactory. Moreover, the references for the propeller’s velocities are within the range of commercial motors for drones, which indicates the applicability of the proposed control scheme in a real vehicle.

### 5.2. Experimental Results

**Figure 14:**
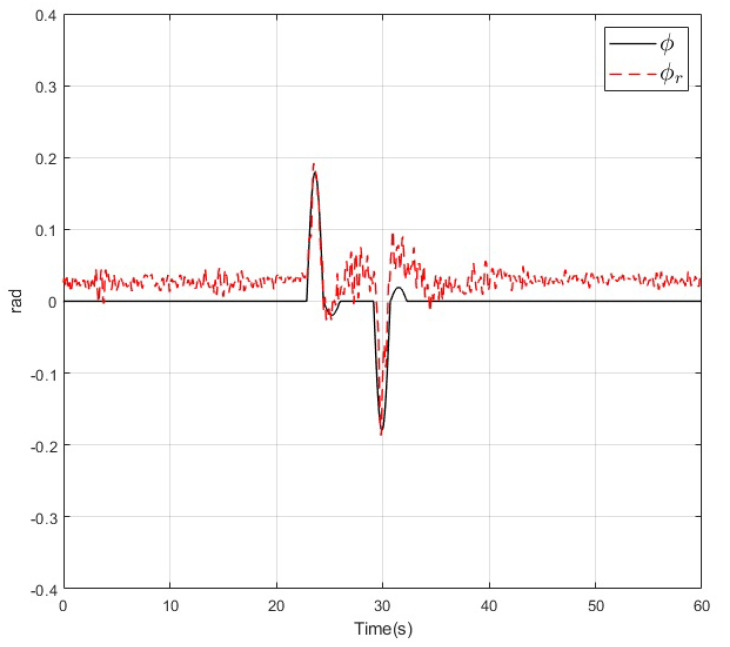
Roll angle (dashed) and its reference (solid) during the real-time experiment.

**Figure 15:**
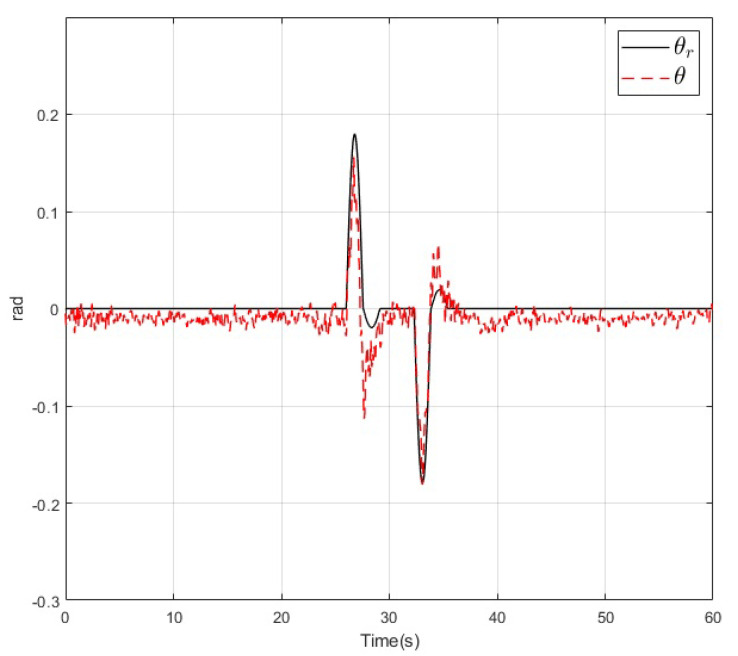
Pitch angle (dashed) and its reference (solid) during the real-time experiment.

**Figure 16:**
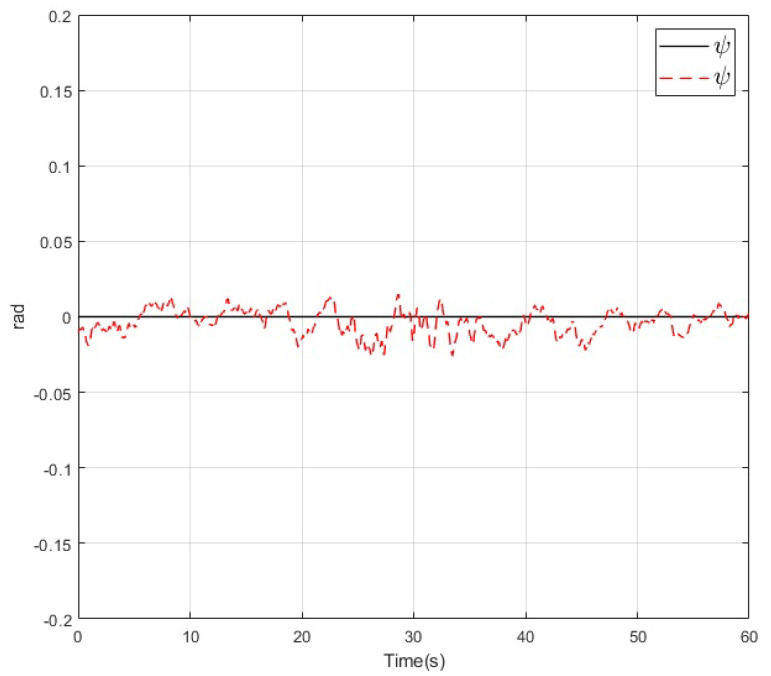
Yaw angle (dashed) and its reference (solid) during the real-time experiment.

**Figure 17:**
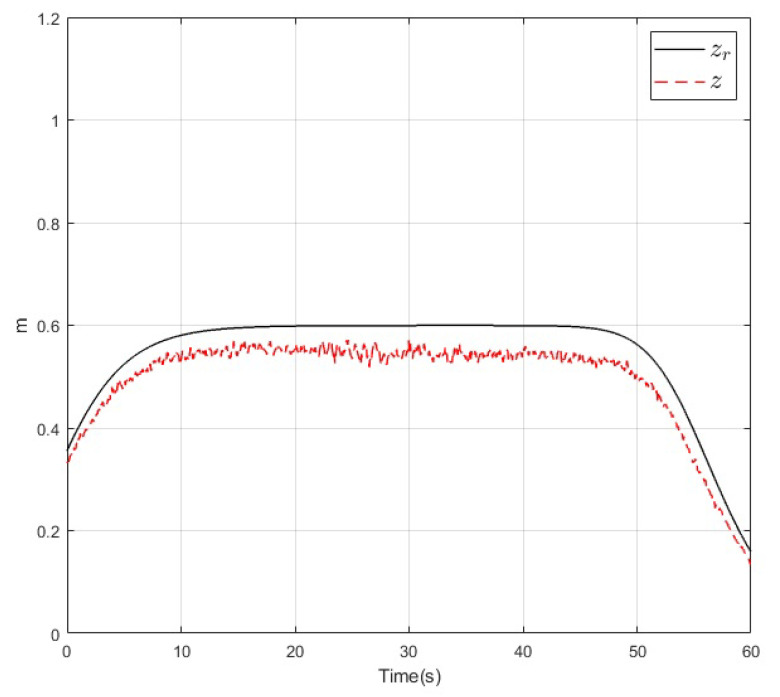
Altitude *z* (dashed) and its reference $z_{r}$ (solid) during the real-time experiment.

The results of the real-time experiments are depicted in the following figures. The roll, pitch, and yaw angles of the vehicle and their references are shown in Figure 14, Figure 15 and Figure 16, respectively. It can be noted that the tracking performance is satisfactory as the convergence of the attitude of the vehicle follows the desired values. Also, a noise component is present in all the measured variables which is normal due to the normal behaviour of the vehicle in flight. On the other hand, the altitude of the vehicle and its desired value are depicted in Figure 17. Its convergence to the reference value can be appreciated as the tracking error variable converges to a small vicinity to zero in a finite time. Finally, a video recording of the real-time experiment can be found in [39] where the performance of the overall control scheme is presented. It is important to mention that the period corresponding to the Figure 14, Figure 15, Figure 16 and Figure 17 corresponds to the period from 0:41 to 1:41 of the reported video.

## 6. Discussion

The proposed control scheme for a UAV based on rotor velocity sensors establishes a solid foundation for developing research related to robust control design, implementation and testing. Also, the developed instrumentation permits to approach real-time thrust computation and wind estimation. On the other hand, although the backstepping technique is widely used and applied in the automatic control field, this work allows to define a comparative reference for more robust and innovative control strategies such as sliding modes, neural networks and adaptive controllers. Moreover, the UAV can be easily adapted to function as a test bench for educational and research purposes related to the design and testing of new control algorithms for multirotors. Finally, the next steps for this research work are related to the design and implementation of robust control schemes based on sliding modes and adaptive control. Also, we are currently exploring fault tolerant controllers which will use the information from the actuators angular velocities and current consumption to detect a failure and reconfigure the controller according to the identified fault.

## 7. Materials and Methods

The manuscript describes the overall structure of the control scheme and the interconnection of the components of the UAV. The proposed angular velocity sensor for the rotors is graphically described in detail so any interested researcher can replicate its implementation. Regarding the software, all the code is available at the repository in [40].

## 8. Conclusions

A control scheme for the trajectory tracking of the attitude and altitude of a multirotor was presented and its effectiveness was demonstrated by means of simulation and real-time experiments. The control strategy is based on the measurements of the angular velocities of the actuators which are open-loop driven BLDC motors. The overall controller consists of two control loops: an outer control loop for the UAV and an inner control loop for the actuators. The UAV controller is designed using the backstepping methodology which assures the closed loop stability of the system. It receives the references for the attitude (Euler angles) and altitude (height) to generate the necessary angular velocities of the actuators to assure the tracking of these references by the vehicle. The actuator control loop receives these necessary angular velocities for the actuators and generates the necessary PWM signals for the BLDC motors to perform the tracking of these angular velocities. This controller is designed based on the PID scheme, which was heuristically tuned to obtain the desired performance.

The simulation results were obtained considering approximated transfer functions for the motors, which were calculated experimentally by means of a proposed test bench. For the real-time experiments, a simple and effective encoder design was implemented based on reflective sensors and a striped band. This is necessary as commercial UAVs do not usually include encoders in the actuator’s axles. Moreover, a conditioning stage for the output of the reflective and the altitude sensors was implemented based on the exponentially weighted moving average (EWMA) filter. The simulation and real-time experiments were satisfactory as the control objectives were fulfilled.

## Figures and Tables

**Table 1:** Multirotor parameters.

Parameter	Description	Value
*m*	Mass of the vehicle	0.85 kg
*g*	Gravity force	9.80665 m/s${}^{2}$
*l*	Arm length	0.1272 m
$C_{T}$	Thrust coefficient	81.7445 × 10${}^{-3}$
$C_{D}$	Drag coefficient	45 × $10^{-3}$
$I_{x}$	Moment of inertia in the *x*-axis	2.14571 × $10^{-3}$ m${}^{2}$
$I_{y}$	Moment of inertia in the *y*-axis	3.28887 × $10^{-3}$ m${}^{2}$
$I_{z}$	Moment of inertia in the *z*-axis	4.94533 × $10^{-3}$ m${}^{2}$
$k_{e}$	Back EMF constant	6.4 × $10^{-4}$
$k_{\tau }$	Torque constant	0.432140018
$R_{a}$	Motor inertia resistance	0.05 Ω
$B_{m}$	Coefficient of viscous friction	341.336 × $10^{-6}$
$J_{m}$	Rotor inertia	1.47106 × $10^{-5}$ kg·m${}^{2}$
$L_{m}$	Inductance	0.012 H

## Abbreviations

The following abbreviations are used in this manuscript:

3D	Three Dimensional
BLDC	Brushless Direct Current
CAD	Computer-Aided Design
EMF	Electro-Mechanical Force
ESC	Electronic Speed Controller
GPS	Global Positioning System
IMU	Inertial Measurement Unit
LQR	Linear Quadratic Regulator
MPC	Model Predictive Control
PID	Proportional-Integral-Derivative
PWM	Pulse-Width Modulation
RMSE	Root Mean Squared Error
UAV	Unmanned Aerial Vehicle
